# Acute Effects of Different Exercise Protocols on the Circulating Vascular microRNAs -16, -21, and -126 in Trained Subjects

**DOI:** 10.3389/fphys.2016.00643

**Published:** 2016-12-26

**Authors:** Patrick Wahl, Udo F. Wehmeier, Felix J. Jansen, Yvonne Kilian, Wilhelm Bloch, Nikos Werner, Joachim Mester, Thomas Hilberg

**Affiliations:** ^1^Institute of Training Science and Sport Informatics, German Sport University CologneCologne, Germany; ^2^Department of Molecular and Cellular Sport Medicine, Institute of Cardiovascular Research and Sport Medicine, German Sport University CologneCologne, Germany; ^3^The German Research Centre of Elite Sport, German Sport University CologneCologne, Germany; ^4^Department for Sports Medicine, University of WuppertalWuppertal, Germany; ^5^Department of Internal Medicine II, Cardiology, Pneumology and Angiology, Medical Faculty, University of BonnBonn, Germany

**Keywords:** miR-16, miR-21, miR-126, endothelial microparticles, angiogenesis

## Abstract

**Aim:** mircoRNAs (miRNAs), small non-coding RNAs regulating gene expression, are stably secreted into the blood and circulating miRNAs (c-miRNAs) may play an important role in cell–cell communication. Furthermore, c-miRNAs might serve as novel biomarkers of the current vascular cell status. Here, we examined how the levels of three vascular c-miRNAs (c-miR-16, c-miR-21, c-miR-126) are acutely affected by different exercise intensities and volumes.

**Methods:** 12 subjects performed 3 different endurance exercise protocols: 1. High-Volume Training (HVT; 130 min at 55% peak power output (PPO); 2. High-Intensity Training (HIT; 4 × 4 min at 95% PPO); 3. Sprint-Interval Training (SIT; 4 × 30 s all-out). c-miRNAs were quantified using quantitative real-time PCR with TaqMan probes at time points pre, 0′, 30′, 60′, and 180′ after each intervention. The expression of miR-126 and miR-21 was analyzed *in vitro*, in human coronary artery endothelial cells, human THP-1 monocytes, human platelets, human endothelial microparticles (EMPs) and human vascular smooth muscle cells (VSMCs). To investigate the transfer of miRNAs via EMPs, VSMCs were incubated with EMPs.

**Results:** HVT and SIT revealed large increases on c-miR-21 [1.9-fold by HVT (cohen's *d* = 0.85); 1.5-fold by SIT (cohen's *d* = 0.85)] and c-miR-126 [2.2-fold by SIT (cohen's *d* = 1.06); 1.9-fold by HVT (cohen's *d* = 0.85)] post-exercise compared to pre-values, while HIT revealed only small to moderate changes on c-miRs-21 (cohen's *d* = −0.28) and c-miR-126 (cohen's *d* = 0.53). c-miR-16 was only slightly affected by SIT (1.4-fold; cohen's *d* = 0.57), HVT (1.3-fold; cohen's *d* = 0.61) or HIT (1.1-fold; cohen's *d* = 0.2). Further *in vitro* experiments revealed that miR-126 and miR-21 are mainly of endothelial origin. Importantly, under conditions of endothelial apoptosis, miR-126 and miR-21 are packed from endothelial cells into endothelial microparticles, which were shown to transfer miR-126 into target vascular smooth muscle cells.

**Conclusion:** Taken together, we found that HVT and SIT are associated with the release of endothelial miRNAs into the circulation, which can function as intercellular communication devices regulating vascular biology.

## Introduction

Endurance training is known to have positive effects on endothelial function and to induce angiogenesis [defined as the formation of new blood vessels from pre-existing vessels, in particular, capillaries (Brown and Hudlicka, 2003)]. However, the molecular mechanisms of these positive effects are still poorly understood.

MicroRNAs (miRNAs) are small non-coding RNAs of 18–22 nucleotides in length, which post-transcriptionally regulate gene expression via mRNA degradation or translational inhibition (Xu et al., 2015). miRNAs can be released into the circulation where they exist in stable forms and play important roles in a wide range of physiological and pathological processes (Ambros, 2004; Mooren et al., 2014). Recent studies suggested that miRNAs are involved in cell–cell-communication (Kosaka et al., 2010) and that circulating miRNAs (c-miRNAs), like hormones and cytokines, mediate gene expression in target cells (Sawada et al., 2013). c-miRNAs have also been suggested as new and alternative biomarkers of muscle damage (Banzet et al., 2013), myocardial infarction (Da Costa Martins and De Windt, 2012), cardiovascular health (Bye et al., 2013), or aerobic performance (Mooren et al., 2014). Alterations of c-miRNAs appear to be rapidly up-regulated during acute physiological stress. Therefore, expression changes of miRNAs may reflect responses to exercise more in detail compared to conventional plasma-based markers, such as creatine-kinase, lactate-dehydrogenase, troponin, C-reactive protein, or interleukin-6 (Baggish et al., 2014; Mooren et al., 2014).

Among the miRNAs involved in the survival, maintenance, and formation of new capillaries, miR-16, -21, and -126 play well-known roles in the control of angiogenesis and vascular integrity (Urbich et al., 2008; Wang et al., 2008; Suárez and Sessa, 2009). miR-21 and miR-126 have been suggested to be pro-angiogenic, whereas miR-16 has been suggested to induce anti-angiogenic processes (Fernandes et al., 2012). Fernandes et al. (2012) reported that in rats, exercise training affects the expression levels of the miRs-16, -21, and -126, thus balancing angiogenic and apoptotic factors like Bcl-2 or VEGF, respectively. Previous studies already investigated the acute effects of different exercise modalities on angiogenic regulating c-miRNAs, recently reviewed by Xu et al. (2015). However, the effects of different exercise intensities, like High-Volume Training (HVT), High-Intensity Training (HIT), and Sprint-Interval Training (SIT), on c-miR-16, -21, and -126 have not been compared yet.

It has already been shown, that HIT and SIT are not only useful tools in elite sports but also in terms of health prevention and rehabilitation (Wahl et al., 2010). HVT and SIT were shown to induce similar increases in muscle oxidative capacity, muscle buffering capacity and glycogen content, despite the distinct differences of these protocols (Gibala et al., 2006). However, only a few studies have focused on endothelial activation, function, and adaptations in response to HVT, HIT, and SIT. In previous studies, we have shown that endurance exercise, independent of intensity, led to decreased endothelial microparticle (EMP) levels and promoted a phosphatidylserine-dependent uptake of EMP into target endothelial cells, which was associated with a protection of target cells against apoptosis (Wahl et al., 2014). EMPs also promote vascular endothelial repair by delivering functional miR-126 into recipient cells (Jansen et al., 2013b). Furthermore, we showed that increasing levels of angiogenic growth factors in the circulation were influenced by exercise intensity (Wahl et al., 2011, 2014).

It has been shown that there is an insufficient oxygen supply in the skeletal muscle during exercise (Shweiki et al., 1992; Jensen et al., 2004; Prior et al., 2004), which might be even more insufficient during intense training (Jones et al., 2015; Oueslati et al., 2016). Additionally, the energy status of the cell and therefore the activation of adenosine monophosphate-activated protein kinase (AMPK) might play a role. AMPK is necessary for hypoxia-induced VEGF mRNA stabilization, but the inhibition of AMPK augmented the VEGF mRNA response to acute exercise (Zwetsloot et al., 2008). This might explain why there are indications that very high-intensity training can actually lead to a negative effect on VEGF levels (Hoier et al., 2013; Gliemann et al., 2015). On the other hand, there is reason to believe, that skeletal muscle adapts to HIT by an increase in capillaries, as oxidative energy metabolism is high, both during exercise and in the recovery phase between exercise bouts (Jensen et al., 2004). Furthermore, mechanical stimuli like total skeletal muscle blood flow, and therefore, shear stress and mechanical stretch are elevated during physical activity, both leading to the elevation of VEGF mRNA and/or protein levels (Brown and Hudlicka, 2003; Prior et al., 2004). The intensity and duration of these mechanical stimuli might also be different during HVT, HIT, and SIT. In the case of a selective miRNA export system, it can be speculated that angiogenic miRNAs are released from endothelial cells into the circulation in response to hypoxia or shear stress, too. Recent reviews by Xu et al. and Altana et al. also consider damaged or apoptotic cells, caused by exercise, as possible sources of c-miRNAs (Altana et al., 2015; Xu et al., 2015). It has been shown that lymphocyte apoptosis occurs in a similar time course as miRNA release (Mooren et al., 2004), underlining that miRNAs could be released from apoptotic cells. However, the potential differential stimuli and associated molecular responses between exercise modalities are still poorly understood and require more investigations.

In order to get a better insight on the influence of exercise on the endothelial cell layer and the underlying mechanisms of angiogenic processes, the aim was to compare the acute effects of different exercise protocols on the circulating vascular microRNAs −16, −21, and −126 in trained subjects.

## Materials and methods

### Subjects

Twelve healthy, non-smoking male triathletes/cyclists (mean ± SD, age: 24.7 ± 3.4 years, weight: 77.5 ± 6.3 kg, height: 183.9 ± 6.3 cm, relative VO_2_max: 64.3 ± 9.7 ml·min^−1^·kg^−1^) participated in the present study. All participants were accustomed to regular training with different exercise intensities, and training for at least 3 years. This cohort of subjects has already been reported in a previous publication (Wahl et al., 2014), and the blood samples of this study were further analyzed according to changes in c-miRNAs expression levels. All subjects were informed orally and in writing of the study's purpose and the possible risks involved before providing written informed consent to participate. The study was approved by the Ethic Committee of the German Sport University Cologne in compliance with the Declaration of Helsinki. Subjects were assigned an anonymous ID during the study.

### Exercise study protocol

Four days before the participation, subjects performed a step test to determine maximal oxygen uptake (VO_2_max) (Zan 600, Zan Messgeräte, Oberthulba, Germany) and maximal peak power output (PPO) in order to determine the intensity for the main experiments. The step test consisted of cycling at ≥80 rpm with an initial workload of 100 W for 5 min and incremental 40-W increases every 5 min until volitional exhaustion was reached.

Subjects participated in three experimental trials on a cycle ergometer (Schoberer Rad Meßtechnik SRM GmbH, Jülich, Germany), each separated by 1 week in a randomized order. In order to have the possibility of comparison with other studies, three commonly used exercise protocols of previous studies were used. The aim was to investigate a broad spectrum of exercise intensities. (1) “HVT”: 2 h at 55% of PPO; (2) “HIT”: 4 × 4 min at 90–95% PPO separated by 3 min of active recovery (45% PPO) each; (3) “SIT”: 4 × 30 s maximal effort (“all out”) separated by 7:30 min of active recovery (45% PPO) each. The HIT and SIT interventions were matched according to total exercise time.

For the “all-out” bouts the ergometer was adjusted to an isokinetic mode set to a cadence of 120 rpm. For HVT and HIT, the ergometer was adjusted to a hyperbolic mode with a self-selected cadence between 80 and 100 rpm. Subjects were instructed to perform the tests in a sitting position on the ergometer. During the all-out bouts, all subjects were vocally encouraged to achieve maximal power output in the same way. For each 30 s all-out exercise bout, mean power output (MPO) was calculated. Before each experimental trial, subjects warmed up for 10 min at an intensity of 50% PPO. During each session, environmental conditions (temperature and humidity) were kept constant and all three tests were carried out at the same time of day in order to prevent diurnal variations. Subjects were not allowed to perform any exercise 48 h prior to all testing. The other days, subjects continued their normal training. The food intake before the test was standardized to the extent that subjects recorded their food intake on the day before the first test, and then were advised to reproduce their diet before each test day. A last snack was allowed 2 h before the test. Thirty minutes after each of the 3 tests, subjects received 500 ml of a low-fat chocolate milk and additional energy-bars. Food intake was adjusted so that energy intake matched the calculated energy expenditure of each trial.

### Measurements

Venous blood samples were collected for the determination of c-miR-16, c-miR-21, c-miR-126 before exercise (pre), and 0 min (0′), 30 min (30′), 60 min (60′), and 180 min (180′) post-exercise. A venipuncture was performed for each sample. Three milliliters of EDTA blood was collected by the Vacutainer blood withdrawal system (Becton Dickinson). Haemoglobin (Hb) and hematocrit (HCT) were analyzed directly with Sysmex KX-21N (CBC) (Sysmex Deutschland GmbH, Norderstedt, Germany). Afterward, the EDTA-blood was centrifuged for 10 min at 1.861 g and 4°C (Rotixa 50, Hettich Zentrifugen, Mühlheim, Germany). The plasma was stored at −80°C until analysis. Methods for blood and lactate analysis have been described previously (Wahl et al., 2014).

### RNA extraction from serum samples and qPCR analyses

The total RNA extraction from EDTA blood serum was performed using a PeqGold Extraction Kit (PeqLab, Erlangen, Germany). Seventy five microliter EDTA-serum was mixed with 40 fmol *Caenorhabditis elegans* miR-39 serum/plasma spike-in control (cel-39; Qiagen Hilden, Germany) and 1 mL PeqGold solution. Total RNA was isolated applying the PeqGold-protocol according to instructions of the manufacturer. The c-miR-16, c-miR-21, and c-miR-126 were then analyzed using a StepOne real-time PCR unit (Life Technologies, Darmstadt, Germany) applying TaqMan miRNA Detection Assays (Life Technologies). Each sample was quantified in duplicate and the mean was recorded. Based on the C_T_ values of the spike-in control the expression rates of the c-miRNAs were calculated. The ratios were normalized to the starting point (pre) of each intervention and the relative changes (x-fold vs. pre) of the c-miRNAs in response to the training were determined. The baseline level variations of the analyzed c-miRNAs were calculated from the pre-values (separated by 1 week) for each intervention. To validate the effect of exercise on the expression the fold change in gene expression was calculated using the equation: $2^{-\Delta {\text{C}}_{\text{T}}}$, where ΔC_T_ = (C_T_, Time × − C_T_, Time 0). Time 0 represents the 1x expression of each gene. The data are presented as the fold change in miRNA expression normalized to the spike-in control (cel-39) and relative to the untreated control/ amount of transcripts at time zero (pre-value). Fold change = $2^{-\Delta \Delta {\text{C}}_{\text{T}}}$ = 2^∧^– ((C_T_, target − C_T_, cel-39) Time × − (C_T_, target − C_T_, cel-39) Time 0). All results were adjusted for changes in plasma volume (PV) [PV changes in percentage of pre-values = [(Hb_pre_/Hb_post_)·(100 − Hct_post_)/[(100 − Hct_pre_) − 1]]·100.

### *In vitro* experiments

Expression of miR-126 and miR-21 was analyzed in human coronary artery endothelial cells (HCAECs), human THP-1 monocytes, human platelets, human endothelial microparticles (EMPs) and human vascular smooth muscle cells (VSMCs). Human coronary artery endothelial cells (HCAEC, PromoCell) were cultured in endothelial cell growth media with endothelial growth media SupplementMix (Promocell) under standard cell culture conditions (37°C, 5% CO_2_). Cells of passage 4–7 were used when 70–80% confluent. EMP were generated from HCAEC as previously described (Jansen et al., 2013a). Briefly, confluent cells were starved by subjecting to media without growth media supplements for 24 h to induce apoptosis of HCAEC. The *in vitro* model of apoptosis was primarily chosen, because Uhlemann et al. (2014) suggested that exercise results in damage to the endothelial cell layer and to create a condition which increases EMPs, with the aim to show that EMP can be transferred to VSMC. *In vivo*, there are of course other stress conditions which lead to the release of EMP. Therefore, the primary focus was not apoptosis itself, but a stress condition which leads to a release of EMPs.

After starvation, the supernatant of apoptotic HCAEC was collected and centrifuged at 1500 g for 15 min to remove cell debris. The supernatant was centrifuged (20,000 g, 40 min.) to pellet microparticles. The obtained EMP was washed in sterile phosphate buffered saline (PBS, pH 7.4), pelleted again at 20,000 g. Pelleted EMP were resuspended in sterile PBS and used freshly. Using flow cytometry and TruCount tubes, EMP were analyzed and the concentration was calculated by the following formula: (number of events for annexin V/number of events in TruCount bead region) × (number of TruCount beads per test/test volume). To analyze microRNA expression in platelets, 20 ml of peripheral blood was collected from our subjects (under resting conditions) in CPT tubes and samples were processed within 15 min of blood collection. Samples were centrifuged at 150 × g for 10 min, and platelet-rich plasma was carefully aspirated and re-centrifuged at 150 × g for 5 min to remove remaining red and white cells. Platelet-rich plasma was centrifuged again at 1500 × g for 10 min to pellet the platelets. The cell pellet was then washed twice with erythrocyte lysis buffer to remove traces of contaminating red blood cells and once with phosphate-buffered saline (PBS). THP-1 monocytes were cultured under standard cell culture conditions (37°C, 5% CO_2_) in RPMI 1640 medium (Invitrogen, CA, USA) containing 10% FBS. Human vascular smooth muscle cells (VSMCs, PromoCell) were cultured in VSMC growth media with appropriate growth media SupplementMix (provitro) under standard cell culture conditions (37°C, 5% CO_2_). Cells of passage 4–7 were used when 70–80% confluent. To investigate the transfer of miRNAs via EMPs, VSMCs were incubated with EMPs (2000 EMP/μl as previously described (Jansen et al., 2012) or PBS as a control for 24 h and miRNA expression was analyzed. For *in vitro* experiments, n represents the number of samples per experiment. Each experiment was performed 3 times.

#### RNA isolation from HCAECs

Total RNA was isolated out of HCAECs, monocytes, platelets, VSMCs, and EMPs by Trizol (Invitrogen) extraction method according to the instruction of the manufacturer. To increase the yield of small RNAs, the RNA is precipitated in ethanol at 20°C overnight with glycogen (Invitrogen). RNA is quantified using Nanodrop spectrophotometer. Then, 10 ng of the total RNA was reverse transcribed using TaqMan microRNA reverse transcription kit (Applied Biosystems, Life technologies, Darmstadt, Germany) according to the manufacturer's protocol. Taqman miRNA assays (Applied Biosystems) were used to measure miR-126 and miR-21 levels on a 7500 HT Real-Time PCR machine (Applied Biosystems). RNU-6 was used as an endogenous control. Delta Ct method was used to quantify relative miRNA expression.

#### Statistics

Statistical analyses of the data were performed by using a statistics software package (Statistica for Windows, 7.0, Statsoft, Tulsa, OK). Descriptive statistics of the data are presented as means ± SE. To assess the effect of the three different interventions on c-miRNAs, a 2-factor [intervention (SIT, HIT, HVT); time (pre-, 0′, 30′, 60′, 180′)] repeated-measures ANOVA with Bonferroni *post-hoc* test was used. Furthermore, cohen's effect size (d) was calculated for the comparison of pre to 0′ values and for the comparison of delta values (0′-pre) between the different exercise protocols (HVT, HIT, SIT). The thresholds for small, moderate, and large effects were defined as 0.20, 0.50, and 0.80, respectively.

## Results

MPO (mean ± SD) of the intervals was 657 ± 54 W during SIT, 278 ± 39 W during HIT, and 164 ± 23 W during HVT. Subjects showed a mean lactate concentration and pH over the whole exercise session of 8.8 ± 2.0 mmol·L^−1^ and 7.26 ± 0.04 during SIT, of 4.4 ± 1.7 mmol·L^−1^ and 7.34 ± 0.03 during HIT, and of 1.0 ± 0.2 mmol·L^−1^ and 7.39 ± 0.01 during HVT, respectively. All parameters differed significantly from each other between the three interventions (Wahl et al., 2014). The mean difference of C_T_ values for the spike in control between all probes was 1.15 ± 0.54 C_T_ while the duplicates of each assay varied 0.09 ± 0.19 C_T_. Prior to all analyses, also the baseline variations for the analyzed miRNAs were calculated as described in the methods section. The baseline expression levels varied as follows: 0.76 ± 0.35 for miR-16, 0.94 ± 0.42 for miR-21; 0.94 ± 0.68 for miR-126. The c-miRNA levels were adjusted to plasma volume changes in order to exclude the effects of exercise-induced hemoconcentration. The changes in PV ranged from −6.2% to +8.5% 0′ and 180′ after SIT, −5.2% to +3.5% 0′ and 180′after HIT and, −0.9% to +5.6% 0′ and 180′ after HVT, respectively.

### c-miR-16

**Figure 1:**
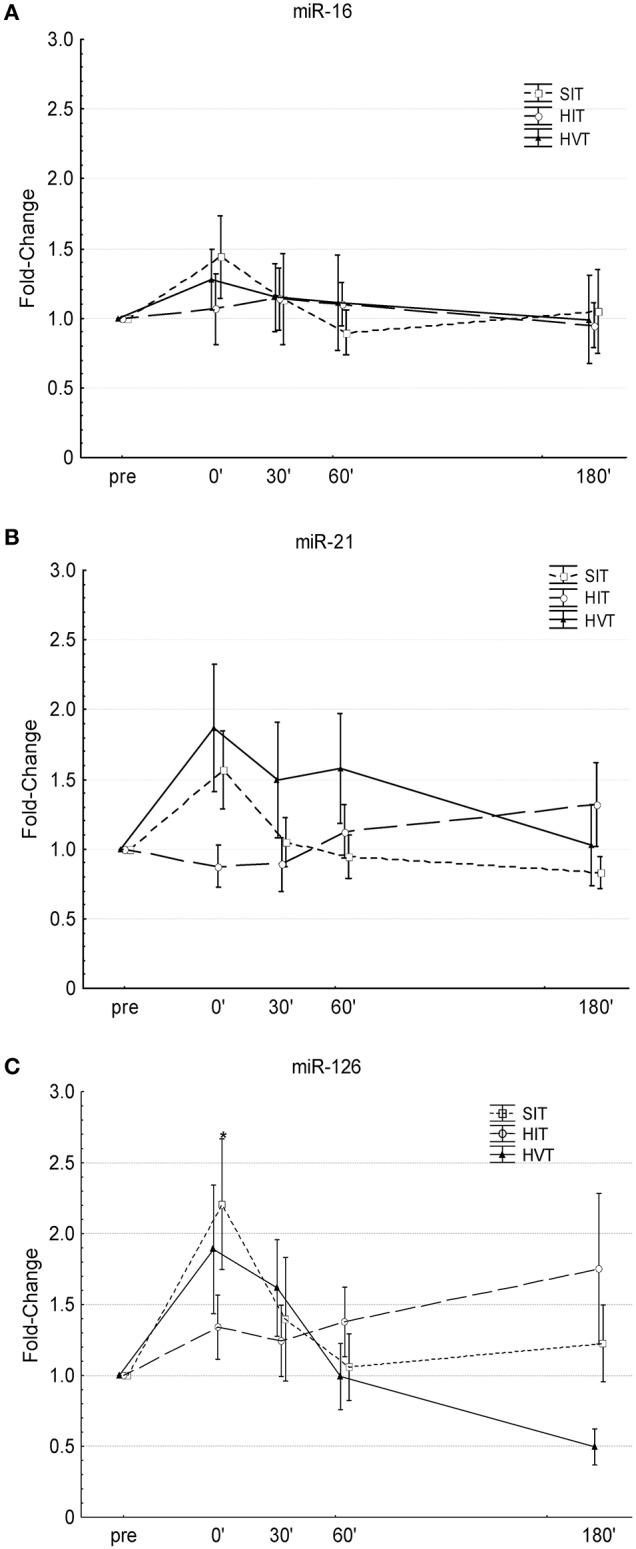
**Changes in circulating miR-16 (A)**, miR-21 **(B)**, and miR-126 **(C)** levels before (pre) and after (0′, 30′, 60′, 180′) each intervention. SIT, sprint-interval training (Squares, dotted line); HIT, high-intensity training (circles, broken line); HVT, high-volume training (triangles, solid line). ^*^Significantly different compared to pre-values of the same intervention (*P* < 0.05); Values are presented as means ± SE.

Overall ANOVA showed no time effect (*p* = 0.09), no intervention effect (*p* = 0.49) and no interaction effect (intervention · time) (*p* = 0.76) (Figure 1A). Small to moderate increases were present from pre-0′ after HIT (cohen's *d* = 0.2), after HVT (cohen's *d* = 0.61) and after SIT (cohen's *d* = 0.57), respectively. Comparison of the delta values (0′-pre) between the different interventions revealed no substantial differences between HIT and HVT and moderate differences between HIT and SIT (cohen's *d* = 0.32), and HVT and SIT (cohen's d = 0.22), respectively.

### c-miR-21

Overall ANOVA showed no time effect (*p* = 0.5), no intervention effect (*p* = 0.53) but a significant interaction effect (intervention · time) (*p* = 0.047). *Post-hoc* analysis revealed no significant changes over time for any of the three condition and no significant differences between conditions (Figure 1B). Small decreases were present from pre-0′ after HIT (cohen's *d* = −0.28), whereas large increases were present from pre-0′ after HVT (cohen's *d* = 0.85) and SIT (cohen's *d* = 0.85). Comparison of the delta values (0′-pre) between the different interventions revealed moderate and large differences between HIT and HVT (cohen's *d* = 0.72), and HIT and SIT (cohen's *d* = 0.89), respectively. No substantial differences were present between HVT and SIT (cohen's *d* = 0.08).

### c-miR-126

Overall ANOVA showed a significant time effect (*p* = 0.007), no intervention effect (*p* = 0.09) and no interaction effect (intervention · time) (*p* = 0.15). *Post-hoc* analysis revealed that SIT significantly increased c-miR-126 0′ post-intervention compared to pre-values (*p* < 0.035) (Figure 1C). Moderate to large increases were present from pre-0′ after HIT (cohen's *d* = 0.53), and after HVT (cohen's *d* = 0.85) and SIT (cohen's *d* = 1.06), respectively. Comparison of the delta values (0′-pre) between the different interventions revealed small differences between HIT and HVT (cohen's *d* = 0.33), and between HVT and SIT (cohen's *d* = 0.32), respectively. Moderate differences were present between HIT and SIT (cohen's *d* = 0.71).

### miR-126 and miR-21 are of endothelial origin and packed into microparticles in apoptotic conditions

**Figure 2:**
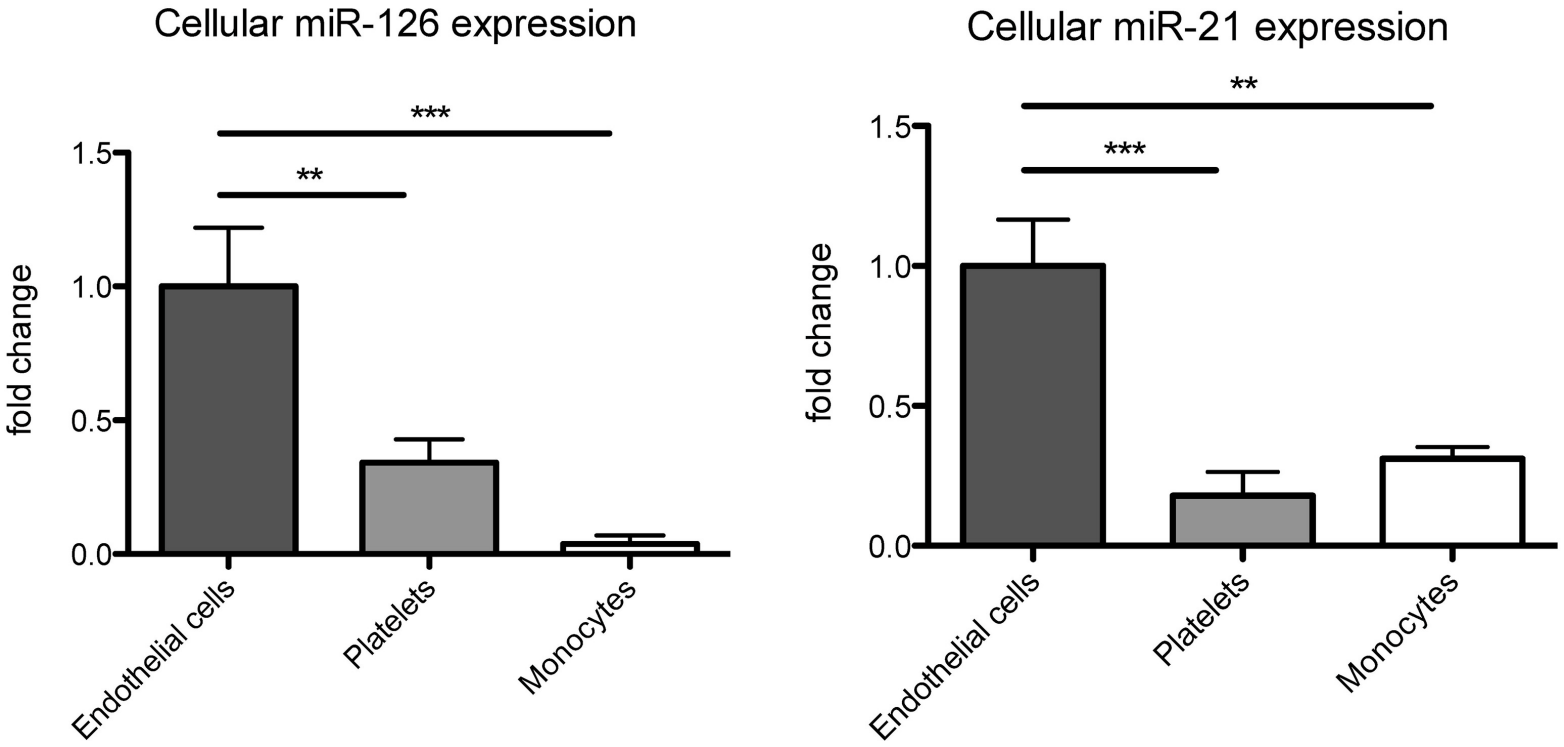
**miR-126 and miR-21 are mainly expressed in endothelial cells**. miR-126 and miR-21 expression were analyzed in human coronary artery endothelial cells, platelets, and THP-1 monocytes. RNU6 was used as endogenous control. *N* = 9–12, ^**^P < 0.01, ^***^P < 0.001.

**Figure 3:**
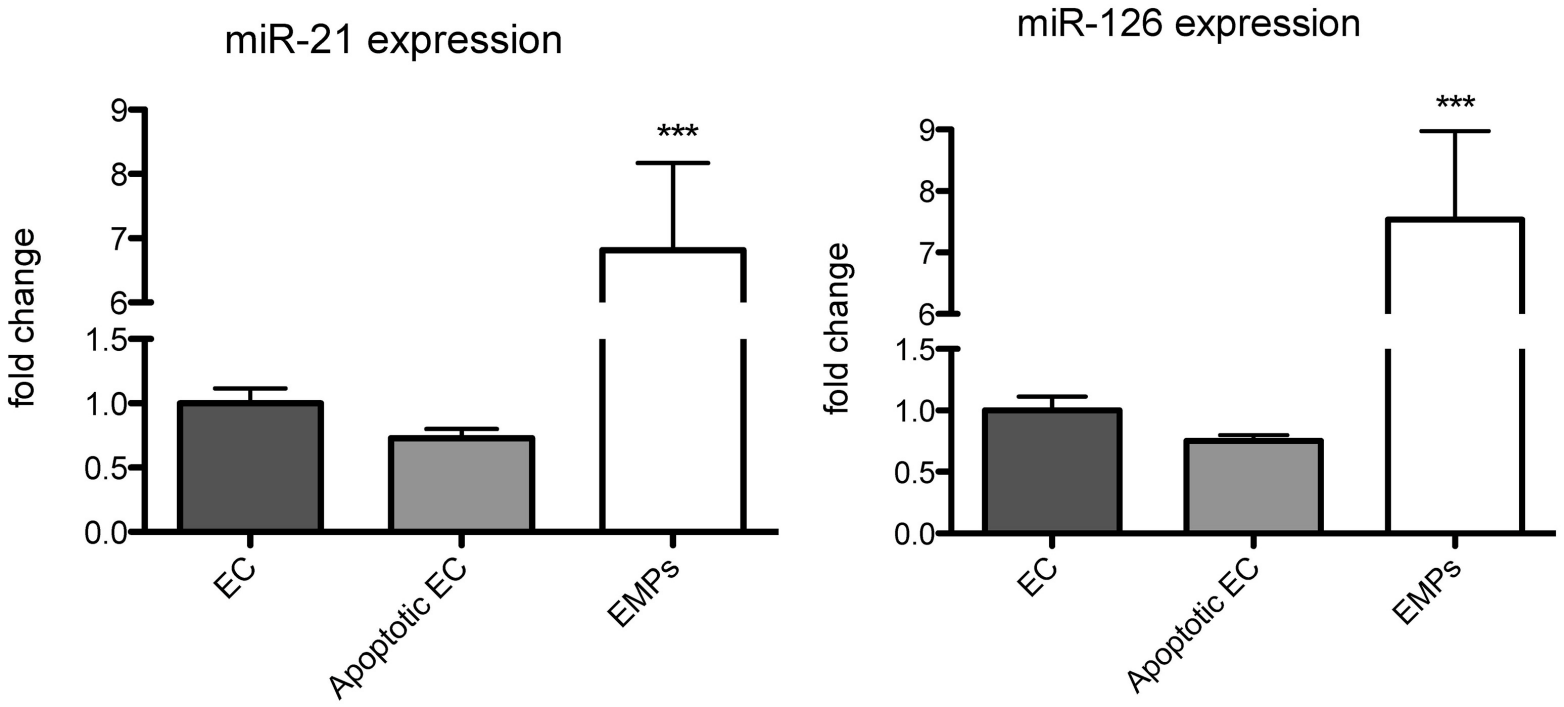
**miR-126 and miR-21 are packed from endothelial cells (EC) into endothelial microparticles (EMPs) under apoptotic conditions**. miR-126 and miR-21 expression was analyzed in ECs, apoptotic ECs, and EMPs. RNU6 was used as endogenous control. *N* = 8–10, ^***^P < 0.001.

As circulating miR-126 and miR-21 showed the strongest regulation after physical exercise, we next aimed to explore their cellular origin. In accordance with previous data (Weber et al., 2010; Jansen et al., 2013b), we found that miR-126 and miR-21 are mainly expressed in endothelial cells compared to monocytes and platelets (Figure 2). As physical exercise was associated with the release of miR-126 and miR-21 into the circulation and was suggested to cause damage to the endothelial cell layer (Uhlemann et al., 2014), we next explored the effect of endothelial apoptosis on endothelial miR-126 and miR-21 expression (Figure 3). Apoptosis of endothelial cells was confirmed using TUNEL-staining, indicating that >60% of cells were apoptotic after 24 h starvation (online Supplemental Figure 1). Terminal deoxynucleotidyl transferase (TdT) dUTP Nick-End Labeling (TUNEL) assay has been designed to detect apoptotic cells that undergo extensive DNA degradation during the late stages of apoptosis (Kyrylkova et al., 2012). Interestingly, we found that both miRNAs were packed from cells into endothelial microparticles, small membrane vesicles, which are released from endothelial cells in conditions of apoptosis (Jansen et al., 2012).

### miR-126 can be transferred from endothelial cells to vascular smooth muscle cells

**Figure 4:**
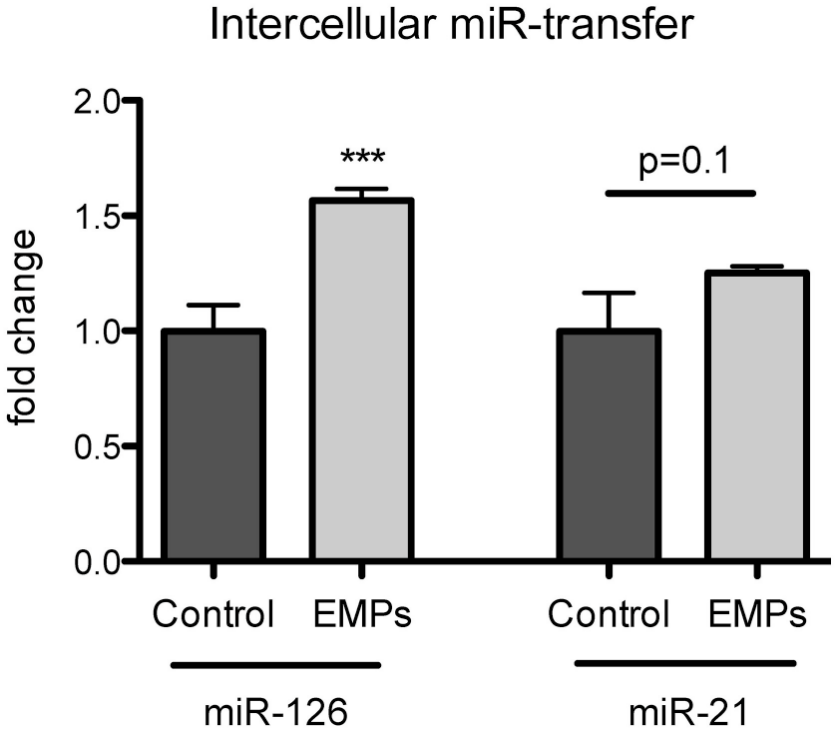
**Endothelial microparticles (EMP)-incorporated miR-126 can be transferred into target vascular smooth muscle cells (VSMCs)**. VSMC were stimulated for 24 h with EMPs or PBS as a control. miR-126 and miR-21 expression was analyzed from stimulated or non-stimulated VSMCs. RNU6 was used as endogenous control. *N* = 7–10, ^***^P < 0.001.

To explore, whether miR-126 and miR-21 might play a role in intercellular communication, we stimulated vascular smooth muscle cells (VSMCs) with EMPs and found that miR-126 expression was significantly, and miR-21 expression slightly increased in target VSMCs after EMP stimulation (Figure 4). These data suggest that miR-126 and miR-21 are not only released in conditions of acute physical exercise but might also function as an intercellular communication device between vascular cells.

## Discussion

In general, there are only a few studies available that have investigated the impact of exercise on miRNA expression either in tissues or in the circulation. Especially different training intensities have not explicitly been taken into account up to now. In the present study, we investigated the effects of three commonly used training protocols of different intensities and volumes on angiogenic regulating c-miRNAs (c-miR-16, c-miR-21, c-miR-126). The major finding of the present study is that SIT and HVT caused the highest increases of c-miR-21 and -126, whereas HIT showed no major influence on these c-miRNAs.

It is important to note that our data were adjusted to the changes in plasma volume, which, to our knowledge, has not been considered in previous studies and which abolish effects that might only reflect concentration changes of c-miRNAs due to plasma volume differences. Sawada et al. (2013) recently criticized that Baggish et al. (2011) did not correct their changes in c-miRNAs for changes in plasma volume, to exclude the effects of exercise-induced hemoconcentration. The corrections for changes in plasma volume are commonly used when analyzing hormones and growth factor in blood samples. However, it has not been clarified so far, whether or not corrections for changes in plasma volume should be used for any plasma-based marker and if changes in plasma volume can also be regarded as a mechanism which leads to changes of c-miRNAs. Without the correction, differences between interventions and changes over time were even more distinct.

The time course of the changes in c-miRNAs resembles the changes of other plasma based markers like hormones and growth factors after such exercise conditions (Wahl et al., 2013). As physiological reactions/changes arise not merely coincidental, acute transient responses might occur to enable the body to cope with different stressors, such as thermal, metabolic, hypoxic, oxidative, and mechanical stress. Different mechanisms of exercise-induced c-miRNAs elevations have been suggested, like cell damage (Uhlemann et al., 2014), *de novo* transcription (Baggish et al., 2014) or activated secretion (Mooren et al., 2014). Cui et al. (2016) suggested that the c-miRNA composition might be a balance between cellular uptake/release and intracellular catabolism and anabolism during exercise. However, the biological significance of exercise-induced c-miRNAs remains elusive.

Aoi et al. (2013) reported that miR-16 is neither affected by acute exercise nor by chronic exercise. In the present study only very weak effects were seen directly at the end of the SIT and HVT protocol, while HIT did not influence the c-miR-16 level at all. miR-16 has been shown to be produced by human endothelial cells and is implicated in suppressing vascular endothelial growth factor (VEGF), VEGF receptor 2 (VEGFR2), basic fibroblast growth factor (bFGF) and fibroblast growth factor receptor 1 (FGF-R1) (Chamorro-Jorganes et al., 2011; Triozzi et al., 2012). miR-16 reduces proliferation, migration, and angiogenic capacity of ECs *in vitro* (Caporali and Emanueli, 2011). As an acute bout of exercise seems not to affect c-miR-16 in trained athletes, one can speculate that the up-regulation of other c-miRNAs, like c-miR-21 or c-miR126 results in a switch of the ratio of these miRNAs and thus promotes the regulatory effects of the up-regulated miRNAs. In our case, the suppressive role of miR-16 on VEGF expression could be overruled by the up-regulation of miR-126 leading to an enhanced VEGF production.

The increases of c-miR-21 after HVT and SIT of the present study are in line with previous studies. After an incremental cycling exercise until exhaustion, c-miR-21 was significantly up-regulated (1.9-fold) immediately after exercise but significantly decreased after 1 h of rest (Baggish et al., 2011). However, Baggish et al. (2011) reported that this responsiveness of c-miR-21 to acute exercise was only present before, but not after sustained training for 90 days in well-trained rowers. This is in line with the finding of Bye et al. (2013) who demonstrated an association between low maximal oxygen consumption and high levels of c-miR-21 expression. This might also explain the results of Nielsen et al. (2014) who found no acute effects of 1 h cycling at 65% Pmax in well-trained subjects on c-miR-21 and decreased resting levels after 12 weeks of endurance training.

Generally, miR-21 regulates several functions relevant to exercise, identifying it as an especially attractive candidate that reflects exercise physiology. In terms of angiogenesis, miR-21 induces angiogenic processes indirectly by enhancing hypoxia inducible factor-1 (HIF-1α) and VEGF expression (Liu et al., 2011), and influences endothelial biology by reducing apoptosis and increasing endothelial nitric oxide synthase activity (Weber et al., 2010). It can be speculated, that especially during all-out exercise bouts, local short-term hypoxic conditions might occur, which could explain the increases of c-miR-21 after SIT. One reason for the increased levels of c-miR-21 after HVT might be prolonged shear stress. In a recent study, Nicoli et al. demonstrated that miR-21 plays a role in the integration of hemodynamics and VEGF signaling during angiogenesis (Nicoli et al., 2010; Weber et al., 2010).

The fact that HVT increased c-miR-126 is in line with previous studies, although these studies investigated even longer exercise durations. Four hours of cycling increased the plasma concentration of c-miR-126 with a maximum 30 min after the start (4.6-fold) and remaining elevated until the end of the test (4.0-fold) (Uhlemann et al., 2014). One hour after finishing the cycling the serum concentration did not differ significantly compared to pre anymore (Uhlemann et al., 2014), which is supported by the present results. Finishing a marathon race resulted in an increase of c-miR-126 in two studies immediately after the race (3.4-fold) (Uhlemann et al., 2014) and (1.9-fold) (Baggish et al., 2014).

miR-126, an endothelial cell-restricted miRNA, is induced by hypoxic stress (Truettner et al., 2013), and regulates vascular integrity and angiogenesis, by enhancing the actions of VEGF and bFGF (Wang et al., 2008). It can be speculated, that hypoxic conditions are more likely to occur during higher intensities, explaining the largest increases of c-miR-126 after SIT (2.2-fold). In previous studies, we were already able to show that SIT also causes the highest increases in VEGF (Wahl et al., 2014). Additionally, Uhlemann et al. (2014) suggested that endurance exercise causes damage of the endothelial cell layer as evident by an increase in c-miR-126. However, this damage might not be caused by shear stress, as Hergenreider et al. (2012) demonstrated that shear stress neither had an influence on miR-126 expression in HUVEC cells, nor on the formation of miR-126 containing vesicles.

Our *in vitro* experiments reveal that miR-126 and miR-21 might play an important role in intercellular communication mechanisms. These findings are in good accordance with the data from Hergenreider et al. (2012) who have also shown that miRNAs could serve as signaling molecules between endothelial and smooth muscle cells. This underlines the potential role of c-miRNAs as signaling molecules between different tissues or organs. However, it has to be considered that c-miRNAs and microvesicle (MV)-encapsulated miRNAs cannot be regarded interchangeably. Wang et al. (2010) who compared profiles of miRNAs in cell-derived vesicles (i.e., exosomes and MVs) with vesicle-free miRNAs (i.e., supernatant fraction after ultracentrifugation) found that miRNA profiles within and outside these vesicles were strikingly different. For miR-126, MVs represent the major plasma compartment, whereas other miRNAs are predominantly found to be transported in the vesicle-free form (Jansen et al., 2014). It might be speculated that miRNAs packed in MVs might be biologically more active and relevant compared with vesicle-free miRNAs. Dependent on the stress condition, the surface of MVs might change, influencing the uptake into target cells, which is not possible for vesicle-free miRNAs. Also, the prognostic value might be different. For miR-126 for example, it has been shown that levels of circulating MVs, but not in plasma have a prognostic relevance for predicting cardiovascular events (Jansen et al., 2014). However, the underlying mechanisms are still unclear. The plasma we analyzed still contained microparticles because the samples were not centrifugated with 100,000 g which would have been necessary to remove the microparticles from the plasma samples (Yuana et al., 2011). Therefore, we cannot clearly distinguish between both fractions of miRNAs and their possible different effects in our *in vivo* experiments. Our *in vitro* experiments are just a model of a stress condition to induce MP release and it can be postulated that mechanisms induced by training are much more multifaceted. Furthermore, a limitation of our study is that downstream markers have not been measured. Further studies have to reveal differences regarding intercellular communication mechanisms between vesicle-free miRNAs and microvesicle (MV)-encapsulated miRNAs and between trained and untrained subjects to better understand the effect of physical exercise on vascular biology.

In summary, our data demonstrate an intensity or volume-dependent regulation of angiogenesis-related c-miRNAs, indicating that the exercise dose may be a crucial trigger. Further *in vitro* experiments revealed that miR-126 and miR-21 are mainly of endothelial origin. Importantly, under conditions of endothelial apoptosis, miR-126 and miR-21 are packed from endothelial cells into endothelial microparticles, which were shown to transfer miR-126 into target vascular smooth muscle cells, which can, therefore, function as intercellular communication devices regulating vascular biology.

## Author contributions

All authors listed, have made substantial, direct and intellectual contribution to the work, and approved it for publication.

## Funding

FJ and NW were supported by Deutsche Forschungsgemeinschaft (WE 4139/8-1). FJ was further supported by the Medical Faculty of the Rheinische Friedrich-Wilhelms-Universität Bonn (BONFOR) and the “Familie Schambach” foundation.

### Conflict of interest statement

The authors declare that the research was conducted in the absence of any commercial or financial relationships that could be construed as a potential conflict of interest.

## Supplementary material

Supplemental Figure 1 **Confirmation of endothelial cell apoptosis by TUNEL-staining**.
