# Supplementary figures and images for: Acute Effects of Different Exercise Protocols on the Circulating Vascular microRNAs -16, -21, and -126 in Trained Subjects
Source: Front Physiol. 2016 Dec 26;7:643. doi: 10.3389/fphys.2016.00643 (PMC5183575; doi:10.3389/fphys.2016.00643)

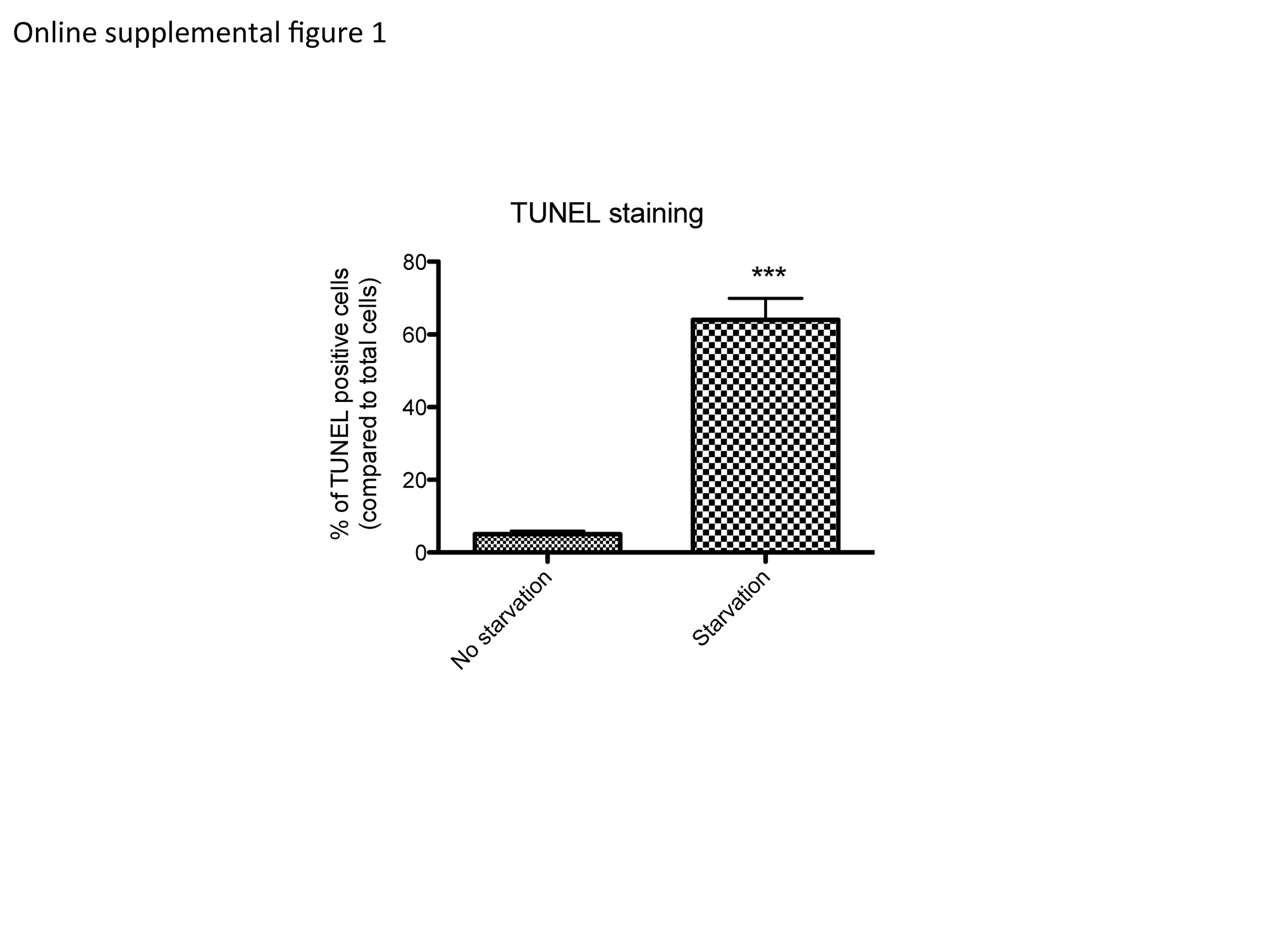

Supplement: Supplemental Figure 1 — Confirmation of endothelial cell apoptosis by TUNEL-staining. [file Image1.TIFF]
